# *Hericium erinaceus* Inhibits TNF-*α*-Induced Angiogenesis and ROS Generation through Suppression of MMP-9/NF-*κ*B Signaling and Activation of Nrf2-Mediated Antioxidant Genes in Human EA.hy926 Endothelial Cells

**DOI:** 10.1155/2016/8257238

**Published:** 2015-12-28

**Authors:** Hebron C. Chang, Hsin-Ling Yang, Jih-Hao Pan, Mallikarjuna Korivi, Jian-You Pan, Meng-Chang Hsieh, Pei-Min Chao, Pei-Jane Huang, Ching-Tsan Tsai, You-Cheng Hseu

**Affiliations:** ^1^Department of Biotechnology and Bioinformatics, Asia University, Taichung 41354, Taiwan; ^2^Institute of Nutrition, College of Biopharmaceutical and Food Sciences, China Medical University, Taichung 40402, Taiwan; ^3^Department of Health and Nutrition Biotechnology, Asia University, Taichung 41354, Taiwan; ^4^Institute of Public Health, China Medical University, Taichung 40402, Taiwan; ^5^Department of Cosmeceutics, College of Biopharmaceutical and Food Sciences, China Medical University, Taichung 40402, Taiwan

## Abstract

*Hericium erinaceus* (HE) is an edible mushroom that has been shown to exhibit anticancer and anti-inflammatory activities. We investigated the antiangiogenic and antioxidant potentials of ethanol extracts of HE in human endothelial (EA.hy926) cells upon tumor necrosis factor-*α*- (TNF-*α*-) stimulation (10 ng/mL). The underlying molecular mechanisms behind the pharmacological efficacies were elucidated. We found that noncytotoxic concentrations of HE (50–200 *μ*g/mL) significantly inhibited TNF-*α*-induced migration/invasion and capillary-like tube formation of endothelial cells. HE treatment suppressed TNF-*α*-induced activity and/or overexpression of matrix metalloproteinase-9 (MMP-9) and intercellular adhesion molecule-1 (ICAM-1). Furthermore, HE downregulated TNF-*α*-induced nuclear translocation and transcriptional activation of nuclear factor-*κ*B (NF-*κ*B) followed by suppression of I-*κ*B (inhibitor-*κ*B) degradation. Data from fluorescence microscopy illustrated that increased intracellular ROS production upon TNF-*α*-stimulation was remarkably inhibited by HE pretreatment in a dose-dependent manner. Notably, HE triggered antioxidant gene expressions of heme oxygenase-1 (HO-1), *γ*-glutamylcysteine synthetase (*γ*-GCLC), and glutathione levels, which may contribute to inhibition of ROS. Increased antioxidant status was associated with upregulated nuclear translocation and transcriptional activation of NF-E_2_ related factor-2 (Nrf2) in HE treated cells. Our findings conclude that antiangiogenic and anti-inflammatory activities of* H. erinaceus* may contribute to its anticancer property through modulation of MMP-9/NF-*κ*B and Nrf2-antioxidant signaling pathways.

## 1. Introduction

The formation of new blood vessel from the preexisting vasculature or angiogenesis is an essential multistep process. However, pathological angiogenesis is a hallmark of several diseases, including cancer, inflammatory diseases, tumor growth metastasis, coronary artery disease, rheumatoid arthritis, and diabetic retinopathy [1, 2]. Degradation of basement membrane by matrix metalloproteinases (MMPs), endothelial cell proliferation/migration, capillary formation, and survival of newly formed blood vessels are the complex sequential steps in completion of angiogenesis. These sequential cascades are tightly regulated by an intricate balance between pro- and antiangiogenic molecules [3, 4]. Among those molecules, tumor necrosis factor-*α* (TNF-*α*), a soluble angiogenic factor produced by many tumors and normal cell lines, plays a key role in regulation of normal and pathologic angiogenesis [4, 5]. It has been indicated that EA.hy926 cells are the best characterized and most frequently used human vascular endothelial cell lines for studying the angiogenesis. Upon stimulation with TNF-*α*, EA.hy926 cells are shown to upregulate intracellular adhesion molecule-1 (ICAM-1), vascular cell adhesion molecule-1 (VCAM-1), and E-selectin expressions that are crucially involved in angiogenesis [6, 7]. Besides, nuclear factor-*κ*B (NF-*κ*B) also plays a vital role in angiogenesis, and expression of MMPs and adhesion molecules are directly coupled with upregulation of NF-*κ*B [8]. In unstimulated condition, NF-*κ*B is localized in the cytoplasm and tethered with its inhibitor protein, I-*κ*B. Upon activation by a variety of external stimuli, including TNF-*α*, the I-*κ*B protein is phosphorylated and degraded in proteasome. This action leads to release of NF-*κ*B, which then translocates to the nucleus and binds to its promoter *κ*B binding site and transcribes a number of genes, including MMPs and adhesion molecules [9, 10]. In addition to this, there is cross talk between NF-*κ*B and nuclear factor (erythroid-2) related factor-2 (Nrf2), which regulates cellular antioxidant status [11].

Activation of antioxidant genes occurs via Nrf2 signaling pathway under stress conditions in order to protect the cells/tissues from oxidative stress [12–14]. Under normal conditions, Nrf2 is sequestered in the cytoplasm by Kelch-like ECH-associated protein 1 (Keap-1). However upon stimulation, Nrf2 translocates into nucleus and recruits the small Maf (sMaf) protein. The Nrf2-sMaf heterodimer then binds to antioxidant response element (ARE), a* cis*-acting DNA regulatory element that activates the promoter region of many genes encoding phase II detoxification enzymes and antioxidants, such as heme oxygenase-1 (HO-1) and glutamate-cysteine ligase (GCLC) [15]. These enzymes exert antioxidant and cytoprotective property by eradicating the toxic free radicals/reactive oxygen species (ROS) in cells.

Fungal mushrooms are valuable foods that are low in calories and high in fibers, minerals, vitamins, and essential amino acids.* Hericium erinaceus* (*H. erinaceus*), an edible and medicinal mushroom, grows on the old or dead broadleaf trees. The fruiting body of* H. erinaceus* has been consumed as a food in Japan/China and used as traditional Chinese medicine (TCM) without any adverse or harmful effects. A large number of studies demonstrated that extracts of* H. erinaceus* have potential therapeutic effects, including antioxidant [16], anticancer [17], anti-inflammatory [18], and stimulating the synthesis of nerve growth factor [19]. So far, several compounds have been isolated from the basidiomata of* H. erinaceus*, such as polysaccharides, erinacines, hericenones, erinapyrones, lectins, proteins, sterols, fatty acid, and esters [20]. Recently, polysaccharide protein HEG-5 purified from* H. erinaceus* has been shown to inhibit growth of gastric tumor cells by promoting cell cycle arrest and apoptosis [21].

Many medicinal mushrooms and herbs are reported to be the rich sources of phytochemicals with chemoprevention potential for various types of human cancers and inflammatory diseases. Because of the critical dependence of human cancer and inflammatory diseases on angiogenesis, therapeutic strategies have been developed targeting various aspects of the angiogenic processes, and many studies demonstrated promising results [22, 23]. On the other hand, cytokines (TNF-*α*) are known to promote angiogenesis by stimulating the proliferation, migration/invasion, and formation of new blood vessels by endothelial cells [6, 24]. In this context, we proposed this study to investigate the antiangiogenic properties of ethanol extracts of* H. erinaceus* (HE) in TNF-*α*-activated human endothelial (EA.hy926) cells. We further explored the underlying molecular mechanism, whether the antiangiogenic property of HE is through the regulation of MMP-9/NF-*κ*B and Nrf2-mediated antioxidant signaling pathways.

## 2. Materials and Methods

### 2.1. Chemicals

Dulbecco's Modified Eagle's Medium (DMEM), fetal bovine serum (FBS), M-199 medium, glutamine, and penicillin-streptomycin-neomycin were obtained from GIBCO BRL (Grand Island, NY). Antibodies against MMP-9, ICAM-1, Nrf2, and I-*κ*B*α* were purchased from Santa Cruz Biotechnology, Inc. (Heidelberg, Germany). Antibodies against anti-NF-*κ*B (p65) were obtained from Cell Signaling Technology Inc. (Danvers, MA). Antibody against HO-1 and *β*-actin was purchased from Abcam (Cambridge, MA). Anti-*γ*-GCLC antibody was obtained from Gene Tex Inc. (San Antonio, TX). All other chemicals were of the highest grade commercially available and supplied either by Merck (Darmstadt, Germany) or Sigma-Aldrich (St. Louis, MO).

### 2.2. Preparation of Ethanol Extracts of* Hericium erinaceus*

The fruit body of* H. erinaceus* was offered by Dr. Chien-Yih Lin from Edible and Medicinal Mushroom Research Center, Asia University, Taiwan. Ethanol extracts from powdered dry fruit bodies were prepared by ultrasonic agitation using 50% ethanol for 15 minutes. The crude extracts were centrifuged at 3000 ×g for 12 min and the supernatant was used for this study. The crude extracts of* H. erinaceus* were concentrated in a rotary evaporation for ethanol and vacuum and freeze dried to form powder. The yield of ethanol extracts of* H. erinaceus* was about 14%. The identified total polyphenol, flavonoid, pentose, and hexose contents from the ethanol extracts of* H. erinaceus* were about 0.08%, 0.01%, 0.8%, and 1.08%, respectively (data not shown). To prepare the stock solution for analysis, the powder samples of* H. erinaceus* were dissolved in 10 mM sodium phosphate buffer (pH 7.4) containing 0.15 M NaCl (PBS) at 25°C. The solution was stored at −20°C before analyses for its antiangiogenic and antioxidant potentials.

### 2.3. Endothelial Cell Culture

The human vascular endothelial cell line (EA.hy926) was grown in DMEM supplemented with 15% FBS, HAT (100 mM sodium hypoxanthine, 0.4 mM aminopterin, and 16 mM thymidine), 1% glutamine, and 1% penicillin-streptomycin-neomycin at 37°C in a 5% CO_2_ humidified incubator. In this study, we used the EA.hy926 cell line because it possessed endothelial characteristics including the formation of tube-like structures [25]. The use of a cell line also allowed us to overcome the difficulty of obtaining larger numbers of uncontaminated primary cells as well as the requirement of expensive growth factors associated with the use of primary endothelial cells. Cultures were harvested and the cell number was determined using a hemocytometer. For all TNF-*α*-stimulated experiments, the supernatant was removed following HE supplementation according to the indicated times, the cells were washed with PBS, and the culture media were replaced with new medium containing 10 ng/mL of TNF-*α* for the indicated time points.

### 2.4. MTT Assay

The effect of HE on cell viability was monitored by the MTT colorimetric assay. EA.hy926 cells at a density of (1 × 10^5^ cells/well) were grown to confluence on 12-well cell culture plates. Cells were pretreated with different concentrations of HE (50–300 *μ*g/mL) for 4 h and then stimulated with TNF-*α* (10 ng/mL) for 24 h. After HE and/or TNF-*α* treatment, the cells were incubated with 400 *μ*L of 0.5 mg/mL MTT in PBS for 2 h. The culture supernatant was removed and resuspended with 400 *μ*L of isopropanol to dissolve the MTT formazan, and the absorbance was measured at 570 nm using ELISA microplate reader (Bio-Tek Instruments, Winooski, VT). The effect of HE on cell viability was assessed as the percent of viable cells compared with the vehicle-treated control cells, which were arbitrarily assigned a viability of 100%. The assay was performed in triplicate at each concentration.

### 2.5. *In Vitro* Wound-Healing Assay

To determine the effects of HE on cell migration, an* in vitro* wound-healing assay was performed. Briefly, EA.hy926 cells at density of 1 × 10^5^ cells/well were cultured with an Ibidi culture-insert on 1% gelatin-coated 12-well plate and incubated with the indicated concentration of HE (50–200 *μ*g/mL for 2 h) in 1% FBS-medium. Cells were then incubated with or without TNF-*α* (10 ng/mL) in fresh medium containing 1% FBS for 24 h. Then the cells were washed twice with PBS, fixed with 100% methanol, and stained with Giemsa Stain solution. The cultures were photographed using optical microscope (200x magnification) to monitor the migration of cells into the wounded area, and the closure of wounded area was calculated using Image-Pro Plus software (Media Cybernetics, Inc., Bethesda, MD).

### 2.6. Endothelial Cell Invasion Assay

Invasion assay was performed using BD Matrigel invasion chambers (BD Biosciences, Bedford, MA). For the invasion assay, 10 *μ*L of Matrigel (25 mg/50 mL) was applied to 8 *μ*m polycarbonate membrane filters, and the bottom chamber of the apparatus contained standard medium. Matrigel is a solubilized basement membrane preparation extracted from the Engelbreth-Holm-Swarm mouse sarcoma, a tumor rich in extracellular matrix proteins. Briefly, the top chambers were seeded with EA.hy926 cells (1 × 10^5^ cells/well) in 500 *μ*L serum-free medium, and the cells were incubated with HE (50–200 *μ*g/mL) for 2 h prior to the addition of 10 ng/mL TNF-*α*. Cells were placed in the bottom chambers (750 *μ*L), which were filled with serum-free medium. Cells were allowed to migrate for 12 h at 37°C. After the incubation period, nonmigrated cells on the top surface of the membrane were removed with a cotton swab. The migrated cells on the bottom side of the membrane were fixed in cold 100% methanol for 8 min and washed twice with PBS. The cells were stained with Giemsa stain solution and then destained with PBS. Images were obtained using an optical microscope (200x magnification); invading cells were quantified by manual counting. Percent inhibition of invading cells was quantified and data expressed as histograms (fold change) by considering untreated cells (control) as 1-fold.

### 2.7. Endothelial Cell Tube Formation Assay

To determine whether AS affected the angiogenic process, tube formation was evaluated using the BD BioCoat angiogenesis system: endothelial cell tube formation assay kit (BD Biosciences, Bedford, MA). In brief, after a treatment with HE (50–200 *μ*g/mL), cells were harvested and seeded in a BD Matrigel Matrix coated 96-well plates with EA.hy926 cells (1 × 10^5^ cells/well) in serum-free medium for 2 h followed by incubating with or without TNF-*α* (10 ng/mL) at 37°C. After 4 h, the capillary networks were photographed using a phase-contrast microscope at 200x magnification; the number of tubes was quantified from three random fields. The percent inhibition was presented as histograms (fold change) by considering untreated cells (control) as 1-fold.

### 2.8. Gelatin Zymography Assay

The activities of MMP-9 released from cells were measured by gelatin zymography protease assays as described previously [23]. Briefly, EA.hy926 cells (1 × 10^5^ cells/well) were seeded into 12-well culture dishes and grown in medium with 15% FBS to a nearly confluent monolayer. The cells were resuspended in medium and then incubated with HE (50–200 *μ*g/mL) for 2 h prior to TNF-*α* (10 ng/mL) incubation. After 24 h, collected media with an appropriate volume (adjusted by vital cell number, 25 *μ*g) were prepared using SDS sample buffer, without boiling or reduction, and were subjected to 1 mg/mL gelatin-8% SDS-PAGE electrophoresis. After electrophoresis, gels were washed with 2.5% Triton X-100 and incubated in a reaction buffer (50 mM Tris-base (pH 7.5), 200 mM NaCl, 5 mM CaCl_2_, and 0.02% Brij 35) at 37°C for 24 h. Then, the gels were stained with Coomassie brilliant blue R-250. The relative MMP-9 activity was quantified by Matrix Inspector 2.1 software.

### 2.9. Preparation of Cell Extracts and Immunoblot Analysis

EA.hy926 cells (5 × 10^5^ cells/6 cm dish) were incubated with various concentrations of HE (50–200 *μ*g/mL for the indicated time) in the presence or absence of TNF-*α* (10 ng/mL) in various time points. After treatment, the cells were detached and washed once in cold PBS and suspended in 100 *μ*L lysis buffer (10 mM Tris-HCl (pH 8), 0.32 M sucrose, 1% Triton X-100, 5 mM EDTA, 2 mM DTT, and 1 mM phenylmethylsulfonyl fluoride). The suspension was put on ice for 20 min and then centrifuged at 15,000 ×g for 20 min at 4°C. Protein content in total, cytoplasmic, and nuclear fractions were determined using a Bio-Rad protein assay reagent, with bovine serum albumin as the standard as described previously [26]. Protein extracts were reconstituted in sample buffer (0.062 M Tris-HCl (pH 6.8), 2% SDS, 10% glycerol, and 5%  *β*-mercaptoethanol), and the mixture was boiled for 5 min. Equal amounts (50 *μ*g) of the denatured proteins were loaded into each lane, separated on 8–15% SDS polyacrylamide gel, and followed by transfer of the proteins to PVDF membranes overnight. Membranes were blocked with 0.1% Tween-20 in Tris-buffered saline containing 5% nonfat dry milk for 20 min at room temperature, and the membranes were reacted with primary antibodies for 2 h. They were then incubated with a horseradish peroxidase-conjugated goat anti-rabbit or anti-mouse antibody for 2 h before being developed using the SuperSignal ULTRA chemiluminescence substrate (Pierce Biotechnology Inc., Rockford, IL). Band intensities were quantified by commercially available software (AlphaEase, Genetic Technology Inc., Miami, FL) and data was presented as histogram.

### 2.10. Immunofluorescence Staining

EA.hy926 cells at a density of 2 × 10^4^ cells/well were cultured in DMEM medium with 15% FBS in an eight-well glass Nunc Lab-Tek chamber and treated with or without HE (50–100 *μ*g/mL for 2 h) in the presence or absence of TNF-*α* (10 ng/mL) for 1 h. Cells were then fixed in 2% paraformaldehyde for 15 min, permeabilized with 0.1% Triton X-100 for 10 min, washed and blocked with 10% FBS in PBS, and then incubated for 2 h with anti-NF-*κ*B (p65) primary antibodies in 1.5% FBS. FITC (488 nm) secondary antibody was incubated for another 1 h in 6% bovine serum albumin. 1 *μ*g/mL 4′,6-diamidino-2-phenylindole (DAPI) was stained for 5 min. Stained cells were washed with PBS and visualized using a confocal microscope at 630x magnification.

### 2.11. Measurement of Intracellular ROS Generation

Production of intracellular ROS was detected by fluorescence microscopy, using DCFH_2_-DA. EA.hy926 cells were plated at a density of 2 × 10^5^ cells/well in a 12-well plate and cultured in DMEM supplemented with 10% FBS. The culture medium was renewed when the cells reached 80% confluence. After HE (50–200 *μ*g/mL) treatment for 2 h, the cells were treated with TNF-*α* (10 ng/mL) for 15 min. Then, the cells were further incubated with 10 *μ*M DCFH_2_-DA in culture medium at 37°C for 30 min. The acetate groups on DCFH_2_-DA were removed by intracellular esterase, trapping the probe inside the cells. Then, the cells were rinsed with warm PBS buffer. The production of ROS was measured as the changes in fluorescence due to the intracellular accumulation of DCF caused by the oxidation of DCFH_2_. The intensity of DCF fluorescence was measured with a fluorescence microscope (200x magnification). Fluorescence intensity was quantified from a square section of fluorescently stained cells, using analysis LS 5.0 soft image solution (Olympus Imaging America Inc., Corporate Parkway Centre Valley, PA), and the fold increase of fluorescence intensity was calculated compared to vehicle-treated control cells.

### 2.12. Determination of Intercellular GSH

GSH levels were determined using the method described by Kamencic and colleagues [27]. Briefly, EA.hy926 cells were treated with HE (100 *μ*g/mL) for 1–8 h, washed twice with PBS, and then incubated with monochlorobimane (2 mM) in the dark for 20 min at 37°C. After two washes with PBS, the cells were solubilized with 1% SDS and 5 mM Tris-HCl (pH 7.4). Fluorescence was measured by fluorescence microplate reader with excitation and emission wavelengths of 380 and 470 nm, respectively. Samples were assayed in triplicate.

### 2.13. Statistical Analyses

All study data were analyzed using an analysis of variance (ANOVA), followed by Dunnett's test for pairwise comparison. Statistical significance was defined as *p* < 0.05 for all tests. Experiment results are presented as mean ± standard deviation (mean ± SD).

## 3. Results

We used human endothelial cell lines, EA.hy926 cells in this study, and the antiangiogenic properties of the ethanol extracts of HE were examined in TNF-*α*-stimulated cells. The molecular mechanisms involved in the inhibition of TNF-*α*-induced angiogenesis were explained based on the experimental evidence.

### 3.1. Effects of HE on EA.hy926 Cell Viability

**Figure 1:**
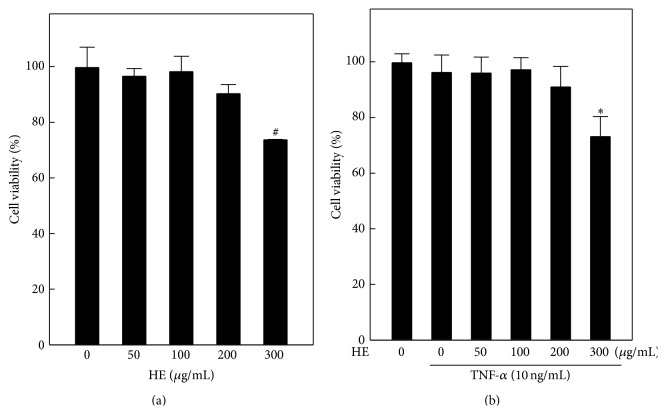
Effect of* Hericium erinaceus* (HE) and/or TNF-*α* on cell viability of human endothelial (EA.hy926) cells. (a) Cells were treated with HE (50–300 *μ*g/mL) for 4 h. (b) Cells were pretreated with HE (50–300 *μ*g/mL) for 4 h and then stimulated with TNF-*α* (10 ng/mL) for 24 h. Cell viability (%) was determined by MTT assay. Results are presented as mean ± SD of three independent assays. ^#^ *p* < 0.05 indicates significant difference compared to control, and ^*∗*^ *p* < 0.05 indicates significant difference compared to TNF-*α* alone treatment.

To determine whether HE treatment could influence cultured endothelial cells, the effect of HE on the viability of EA.hy926 cells with or without TNF-*α*-stimulation was examined by MTT assay. We found that HE pretreatment up to 200 *μ*g/mL concentration did not affect the cell number without or with TNF-*α*-stimulation for 24 h (Figures 1(a) and 1(b)). No distinct cellular or morphological changes were detected after a 4 h incubation of HE at the concentration < 200 *μ*g/mL. However, cells exposed to 300 *μ*g/mL of HE for 4 h showed significantly decreased proliferation. The decreased viability of EA.hy926 cells with higher HE concentrations was observed in both stimulated and unstimulated conditions (Figures 1(a) and 1(b)). Therefore, the noncytotoxic concentration of HE (i.e., ≤200 *μ*g/mL) was used for all experiments, and its potentials were evaluated on TNF-*α*-induced angiogenesis.

### 3.2. HE Inhibits TNF-*α*-Induced Migration and Invasion of Endothelial Cells

**Figure 2:**
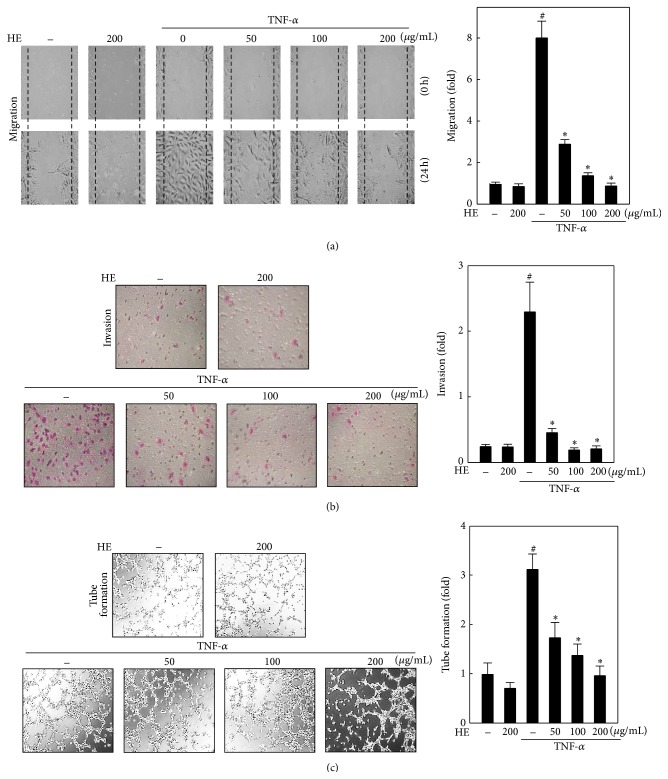
HE inhibits TNF-*α*-induced migration, invasion, and capillary-like tube formation in EA.hy926 cells. (a) Cells were pretreated with HE (50–200 *μ*g/mL) for 2 h. Subsequently, cells were scratched and then stimulated with or without TNF-*α* (10 ng/mL) for 24 h. Migration was observed using an optic microscope, at a 200x magnification, and the closure of area was calculated using commercially available software. (b) Cells were pretreated with HE (50–200 *μ*g/mL) for 2 h followed by incubation with or without TNF-*α* (10 ng/mL) for 12 h. Photomicrographs of cells invading under the membrane. The inhibitory percentage of invading cells was quantified and fold change was presented by considering untreated cells (control) as 1-fold. Invasiveness was determined by counting cells in three microscopic fields per sample. (c) Cells were pretreated with HE (50–200 *μ*g/mL) for 2 h and then collected and replaced on Matrigel-coated plates at a density of 1 × 10^5^ cells/well and incubated in the absence or presence of TNF-*α* (10 ng/mL). After 4 h, the tube formation was determined using a phase-contrast microscope at 200x magnification. The capillary networks were photographed, and the number of tubes was quantified from three random fields. Results are presented as mean ± SD of three independent assays; ^#^ *p* < 0.05 indicates significant difference compared to control, and ^*∗*^ *p* < 0.05 indicates significant difference compared to TNF-*α* alone treatment.

Endothelial cells migration and invasion through the basement membrane is an important step in formation of new capillary tubes. To determine the effects of HE on endothelial cell migration* in vitro*, confluent monolayers of EA.hy926 cells were incubated with or without HE in the presence or absence of TNF-*α* (10 ng/mL) for 24 h. As shown in Figure 2(a), TNF-*α* significantly increased the migration of endothelial cells, whereas addition of HE (50–200 *μ*g/mL) dose-dependently decreased the TNF-*α*-induced migration of EA.hy926 cells. The effect of HE on invasiveness of EA.hy926 cells was evaluated using the Boyden chamber assay, which allowed to determine the ability of cells to pass through a layer of extracellular matrix on a Matrigel-coated filter. The results showed that invasiveness of EA.hy926 cells was significantly enhanced upon TNF-*α* exposure for 12 h. But HE incubation attenuated the TNF-*α*-induced invasion in a dose-dependent manner (Figure 2(b)). Regardless of HE effects on stimulated cells' migration and invasion ability, HE alone (200 *μ*g/mL) did not affect either migration or invasion of unstimulated endothelial cells.

### 3.3. HE Inhibits TNF-*α*-Induced Tube Formation by EA.hy926 Cells

During angiogenesis vascular maturation is characterized by the formation of tubular structures by capillary endothelial cells [28]. In this study, the influence of HE treatment on TNF-*α*-induced formation of capillary-like structure was examined by tube formation assay. Upon TNF-*α* exposure for 4 h, EA.hy926 cells aligned into cords on the Matrigel, which is proportionate to the enhanced formation of tube-like structures (Figure 2(c)). Similar to the migration and invasiveness of endothelial cells, treatment with different concentrations of HE (50–200 *μ*g/mL) resulted in a dose-dependent inhibition of TNF-*α*-induced tube formation. In addition, HE alone (200 *μ*g/mL) tends to inhibit tube formation of endothelial cells.

### 3.4. HE Suppresses TNF-*α*-Induced MMP-9 Activity and Expression in EA.hy926 Cells

**Figure 3:**
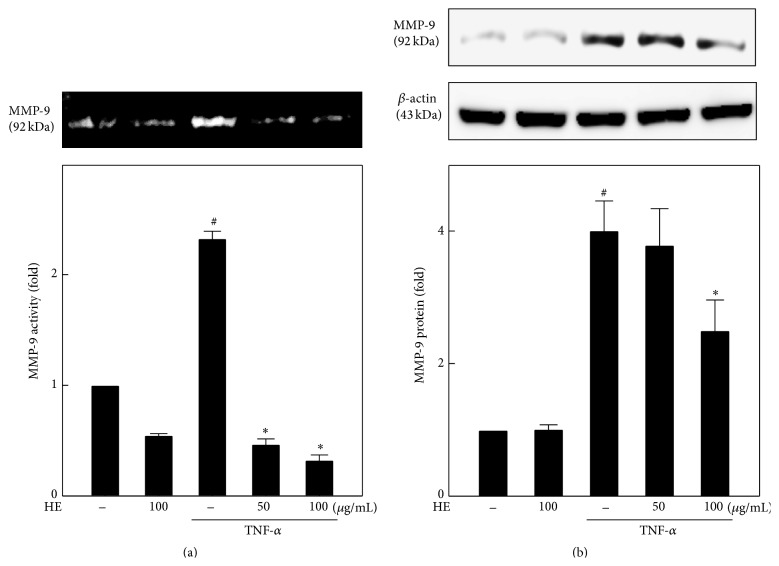
HE suppresses MMP-9 activity and expression in TNF-*α*-stimulated EA.hy926 cells. Cells were pretreated with 50 and 100 *μ*g/mL HE for 2 h and then stimulated with TNF-*α* (10 ng/mL) for 24 h. (a) An equal amount (50 *μ*g) of conditioned culture media from each sample was subjected to gelatin zymography. The relative density of MMP-9 bands was measured by commercially available quantitative software. (b) An equal amount (50 *μ*g) of total lysate from each sample was resolved by 10% SDS-PAGE with *β*-actin as a control. The relative changes in protein bands were measured, the control being 1-fold, as shown just below the gel data. Results are presented as mean ± SD of three independent assays; ^#^ *p* < 0.05 indicates significant difference compared to control, and ^*∗*^ *p* < 0.05 indicates significant difference compared to TNF-*α* alone treatment.

It has been reported that gelatinase and collagenase MMPs are involved in the angiogenic process [29]. Therefore, we performed gelatin zymography and western blot assays to determine the changes in MMP-9 activity and protein expression. EA.hy926 cells were treated with or without HE (50 and 100 *μ*g/mL) in the presence or absence of TNF-*α* in serum-free medium. We found that cells exposed to TNF-*α* for 24 h substantially increased the MMP-9 activity (Figure 3(a)). However, cells coincubated with HE contributed to suppress the TNF-*α*-induced MMP-9 secretion in a dose-dependent manner. HE alone (100 *μ*g/mL) also inhibited the MMP-9 activity of endothelial cells (Figure 3(a)). Similar to the results of MMP-9 activity, MMP-9 protein expression was also upregulated with TNF-*α* alone in EA.hy926 cells. Here it is interesting to note that only 100 *μ*g/mL of HE cotreatment suppressed the TNF-*α*-induced MMP-9 overexpression, but not with 50 *μ*g/mL HE cotreatment (Figure 3(b)). Besides, HE treatment (100 *μ*g/mL) alone had no effect on MMP-9 protein expression in endothelial cells (Figure 3(b)).

### 3.5. HE Attenuates the TNF-*α*-Induced ICAM-1 Expression in EA.hy926 Cells

**Figure 4:**
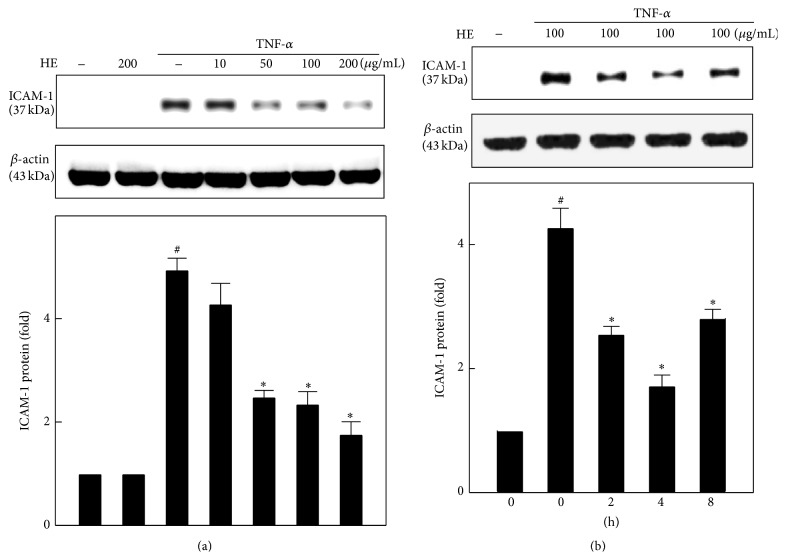
HE inhibits TNF-*α*-induced ICAM-1 expression in EA.hy926 cells. (a) Cells were harvested after pretreatment with the indicated concentration of HE (10–200 *μ*g/mL) for 2 h in the absence or presence of TNF-*α* (10 ng/mL) for 4 h. (b) Cells were pretreated with HE (100 *μ*g/mL) for 2–8 h in the presence or absence of TNF-*α* (10 ng/mL for 4 h). An equal amount (50 *μ*g) of total lysate from each sample was resolved by 12% SDS-PAGE with *β*-actin as a control. Relative changes in protein bands were measured using densitometric analysis. Results are presented as mean ± SD of three independent assays; ^#^ *p* < 0.05 indicates significant difference compared to control, and ^*∗*^ *p* < 0.05 indicates significant difference compared to TNF-*α* alone treatment.

We determined the effect of HE on ICAM-1 protein expression in EA.hy926 cells in the presence or absence of TNF-*α* exposure for 4 h. Western blot data presented in Figure 4(a) demonstrated that cells exposed to TNF-*α* significantly increased the ICAM-1 protein levels, whereas HE treatment (10–200 *μ*g/mL) substantially suppressed the TNF-*α*-induced ICAM-1 overexpression in a dose-dependent manner. Next we conducted the time-dependent studies (0–8 h) to examine the HE-mediated inhibition of ICAM-1 against TNF-*α*-induced upregulation (Figure 4(b)). We found that increased ICAM-1 protein upon TNF-*α* exposure was downregulated by HE at 2, 4, and 8 h after treatment, but the maximum reduction was found at 4 h after treatment in endothelial cells (Figure 4(b)).

### 3.6. HE Suppresses TNF-*α*-Stimulated NF-*κ*B Activation and I-*κ*B*α* Degradation in EA.hy926 Cells

**Figure 5:**
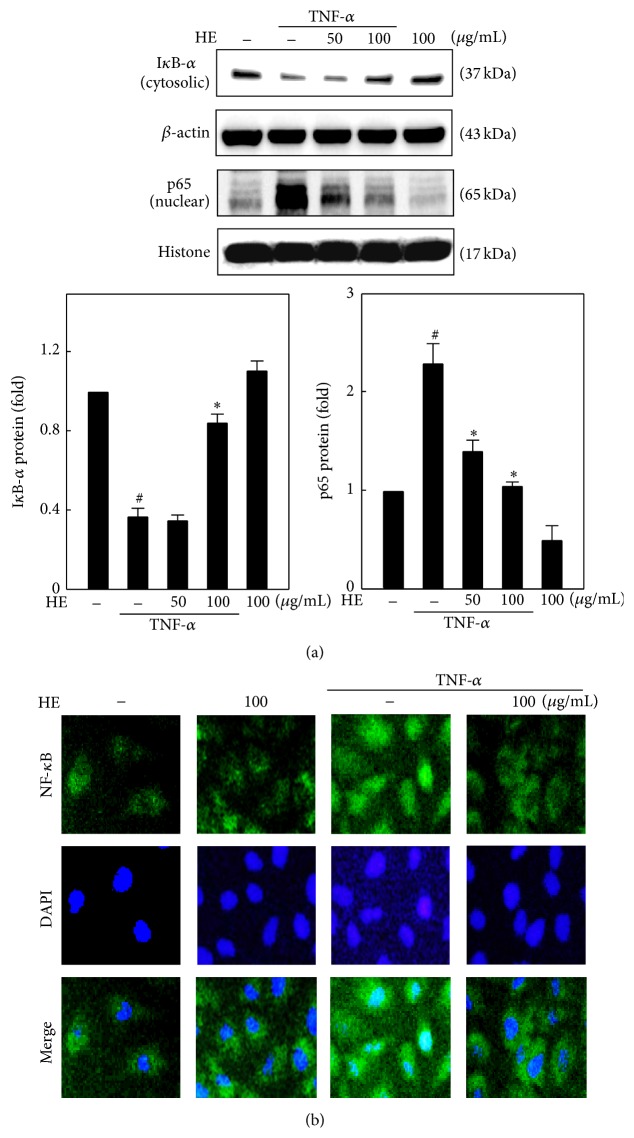
HE downregulates NF-*κ*B nuclear translocation through the suppression of I-*κ*B degradation in TNF-*α*-activated EA.hy926 cells. (a) Western blot was performed to monitor the cytosolic I-*κ*B*α* and nuclear p65 protein expressions. Cells were treated with indicated concentrations of HE (50–100 *μ*g/mL) for 2 h in the presence or absence of TNF-*α* (10 ng/mL) for 1 h. An equal amount (50 *μ*g) of cytosolic or nuclear lysate from each sample was resolved by 12% SDS-PAGE with *β*-actin as a control. Relative changes in protein bands were measured using densitometric analysis. (b) Immunofluorescence staining shows the changes of nuclear translocation of NF-*κ*B. Cells were grown in chamber slides that were exposed to HE (100 *μ*g/mL) for 2 h in the presence or absence of TNF-*α* (10 ng/mL) for 1 h, fixed, and permeabilized. Then the cells were incubated with anti-P65 antibody followed by FITC-labeled secondary antibody. Cells were stained with DAPI (1 *μ*g/mL) for 5 min and examined by fluorescence microscopy (magnification ×200). Results are presented as mean ± SD of three independent assays; ^#^ *p* < 0.05 indicates significant difference compared to control, and ^*∗*^ *p* < 0.05 indicates significant difference compared to TNF-*α* alone treatment.

NF-*κ*B activation is a critical event for TNF-*α*-induced MMP and adhesion molecule activation/expression [8]. Since phosphorylation and subsequent proteasomal degradation of I-*κ*B*α* is a critical step for the export of NF-*κ*B subunits to the nucleus, we further examined the effect of HE (50 and/or 100 *μ*g/mL for 1 h) on TNF-*α*-induced I-*κ*B*α* degradation and NF-*κ*B activation in EA.hy926 cells. Compared to control, TNF-*α* exposure remarkably reduced the amount of I-*κ*B*α* protein, whereas HE treatment prevented the TNF-*α*-induced I-*κ*B*α* degradation in a dose-dependent manner (Figure 5(a)). Western blot analysis with nuclear fraction confirmed that TNF-*α* increased the accumulation of NF-*κ*B (p65). However TNF-*α*-induced nuclear translocation of p65 was dose-dependently inhibited by HE treatment (Figure 5(a)). Images from immunofluorescence assay illustrated that NF-*κ*B (p65) was tethered in cytoplasm of untreated control cells, but nuclear NF-*κ*B was spontaneously increased after TNF-*α*-stimulation (Figure 5(b)). Consistent with western blot data, HE treatment (100 *μ*g/mL) prevented TNF-*α*-induced nuclear translocation of NF-*κ*B in EA.hy926 cells (Figure 5(b)). These findings imply that HE downregulates the TNF-*α*-induced NF-*κ*B activation followed by the suppression of I-*κ*B degradation.

### 3.7. HE Inhibits TNF-*α*-Induced Intracellular ROS Generation in EA.hy926 Cells

**Figure 6:**
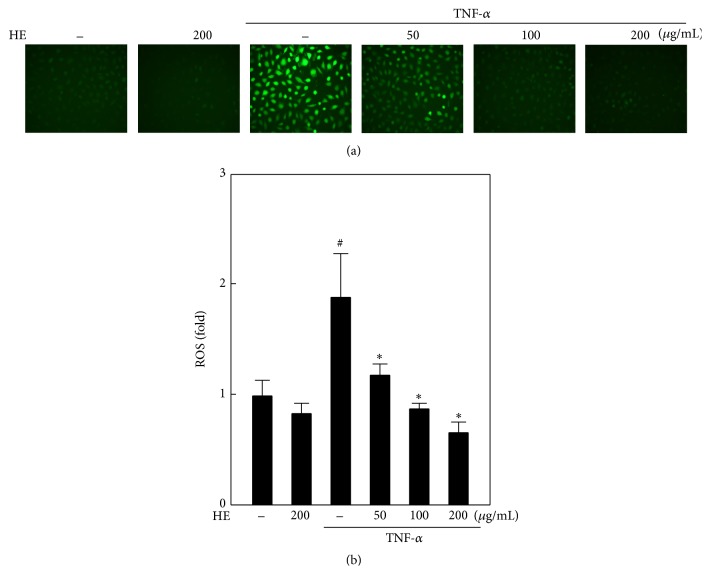
HE inhibits TNF-*α*-induced ROS production in EA.hy926 cells. Cells were preincubated with or without HE (50–200 *μ*g/mL) for 2 h and then stimulated with TNF-*α* (10 ng/mL) for 15 min. (a) Intracellular ROS levels were measured using a DCFH_2_-DA fluorescence microscope (200x magnification). The nonfluorescent cell membrane-permeable probe, DCFH_2_-DA, was added to the culture medium at a final concentration of 10 *μ*M. DCFH_2_ reacts with cellular ROS and is metabolized into fluorescent DCF. (b) The fluorescence intensity of DCF-stained cells was quantified as a percentage, using Olympus soft image solution software. Results are presented as mean ± SD of three independent assays; ^#^ *p* < 0.05 indicates significant difference compared to control, and ^*∗*^ *p* < 0.05 indicates significant difference compared to TNF-*α* alone treatment.

Next, we used a DCFH_2_-DA fluorescent staining method to determine whether HE could be able to inhibit the TNF-*α*-induced ROS generation in EA.hy926 cells. As shown in Figures 6(a) and 6(b), intracellular ROS generation was dramatically increased upon TNF-*α*-stimulation for 15 min. Increased DCF fluorescence intensity is directly proportionate to the amount of ROS generation in the cells. Nevertheless, HE treatment (50–200 *μ*g/mL) remarkably suppressed the TNF-*α*-induced excessive ROS in a dose-dependent manner (Figures 6(a) and 6(b)). These findings reveal that HE may protect the EA.hy926 endothelial cells from TNF-*α*-induced oxidative stress by inhibition and/or scavenging of ROS.

### 3.8. HE Upregulates HO-1 and *γ*-GCLC Expression via Nrf2 Activation in EA.hy926 Cells

**Figure 7:**
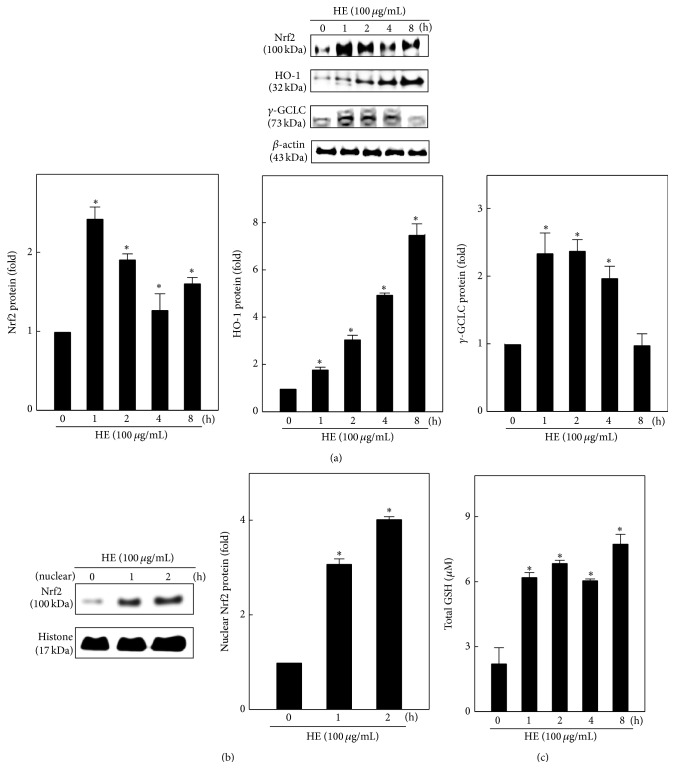
HE upregulates the Nrf2-mediated antioxidant genes in EA.hy926 cells. (a) Western blotting was performed to monitor the Nrf2, HO-1, and *γ*-GCLC protein using specific antibodies. Cells were treated with HE (100 *μ*g/mL) 1–8 h. The total cell lysate was subjected to western blotting. (b) Western blot data shows the changes of Nrf2 nuclear translocation. Cells were treated with HE (100 *μ*g/mL) 1 or 2 h. An equal amount (50 *μ*g) of nuclear lysate from each sample was resolved by 8–15% SDS-PAGE with *β*-actin as a control. The relative changes in the intensities of the protein bands were measured by densitometry. (c) The increasing amount of total GSH was measured using commercially available EIA kit. Cells were incubated with HE (100 *μ*g/mL) for 1–8 h. One of the typical results from three independent experiments is shown. Results are presented as mean ± SD of three independent assays; ^*∗*^ *p* < 0.05 indicates significant difference in comparison to control cells.

We hypothesized that the protective effects of HE against TNF-*α*-induced ROS generation are possibly due to the induction of antioxidant genes, such as HO-1 and *γ*-GCLC, and its transcription factor Nrf2. To affirm this phenomenon, cells were treated with HE (100 *μ*g/mL) for 1–8 h, and changes in Nrf2, HO-1, and *γ*-GCLC expressions were monitored by western blot. As expected, HE treatment significantly increased the Nrf2, HO-1, and *γ*-GCLC expressions in a time-dependent manner (Figure 7(a)). The maximum elevation of Nrf2 at 1 h, HO-1 at 8 h, and *γ*-GCLC at 1/2 h was observed in cells after HE treatment. HE-mediated induction of HO-1 and *γ*-GCLC may be due to the nuclear translocation and transcriptional activation of Nrf2. Therefore, we estimated the Nrf2 in nuclear fractions of EA.hy926 cells. Western blot data showed that HE treatment (100 *μ*g/mL) significantly increased the nuclear Nrf2 levels (Figure 7(b)). In the present study, we observed that HE upregulates *γ*-GCLC expression in EA.hy926 cells, which is essential for the* de novo* synthesis of GSH. Therefore, next we examined whether HE treatment could promote the GSH synthesis in EA.hy926 cells. As shown in Figure 7(c), the intracellular GSH level was significantly increased by HE treatment. These results suggest that HE might promote the induction of HO-1 and *γ*-GCLC antioxidant genes and GSH production followed by transcriptional activation of Nrf2 in EA.hy926 cells.

## 4. Discussion

**Figure 8:**
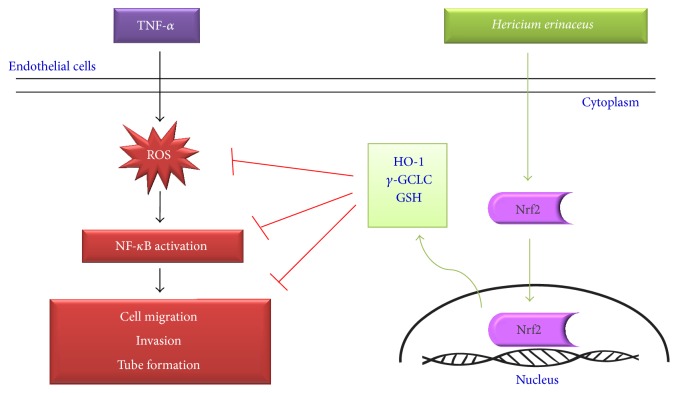
Schematic representation of HE antiangiogenic and anti-inflammatory properties through induction of Nrf2-mediated antioxidant genes in human endothelial cells.

This is the first study to demonstrate the antiangiogenic, anti-inflammatory, and antioxidant properties of* H. erinaceus* (HE) extracts in cytokine-activated human endothelial EA.hy926 cells. Underlying molecular mechanisms behind these therapeutic effects were explained with novel experimental evidence. Our findings showed that the noncytotoxic concentrations of HE pretreatment substantially inhibited the endothelial cell migration, invasion, and tube formation after challenge with TNF-*α*, which implies the potent antiangiogenic property of HE. In addition, TNF-*α*-induced overexpression of MMP-9 and ICAM-1 were remarkably inhibited by HE in a dose-dependent manner. This phenomenon was accompanied by suppression of transcriptional activation of NF-*κ*B (p65) and subsequent inhibition of I-*κ*B degradation. Furthermore, cells incubated with HE increased the expressions of antioxidant genes, including HO-1 and *γ*-GCLC via Nrf2 signaling pathway. This was further confirmed by suppression of intracellular ROS accumulation in HE pretreated cells against TNF-*α*-stimulation. HE-induced downregulation of NF-*κ*B activation and upregulation of antioxidant genes may collectively attribute to suppression of TNF-*α*-induced angiogenesis in endothelial cells (Figure 8). Because tumor epithelial cells depend on angiogenesis to provide nutrients for their growth and survival, it is plausible that antiangiogenic effect may play a primary role in mediating the cancer chemopreventive activity [30]. Moreover, the pharmacology of many anti-inflammatory drugs revealed that at least part of their efficacy is attributable to their antiangiogenic effects [31]. In light of this, HE may be considered as a potent antiangiogenesis candidate for developing of novel drugs to treat diseases with impaired inflammation and cancer.

Angiogenesis is an invasive process that requires proteolysis of the extracellular matrix and proliferation and migration of endothelial cells, as well as synthesis of new matrix components. The MMPs have been shown to influence angiogenesis by degrading matrix molecules and by activating or liberating growth factors, including cytokines [3]. Among several members, MMP-9 is important in endothelial cell morphogenesis and capillary formation [32]. The release of MMP-9 from endothelial cells represents an important step in neovascularization, because this major extracellular matrix proteolytic enzyme is secreted when endothelial sprouting takes place, thus enhancing cell migration across the extracellular matrix and tube-like structure formation [29]. In our study, release of MMP-9 upon TNF-*α*-stimulation was found to be suppressed by HE treatment in endothelial cells. This phenomenon implies that antiangiogenic property of HE is closely associated with decreased endothelial cell MMP-9 activity. Both* in vivo* and* in vitro* studies clearly demonstrated that absolute lack/inhibition of MMP-9 can reduce the cell-cell interaction and prevent the formation of new capillary network [32]. HE-induced suppression of MMP-9 in our study was accompanied with inhibition of endothelial cell migration, invasion, and tube formation, which emphasizes the critical role of MMP-9 and HE in tumor treatment.

Expression of adhesion molecules, including ICAM-1, VCAM-1, and endothelial leukocyte adhesion molecule-1 (ELAM-1), on endothelial cells is critical for tumor cell invasion and metastases. Therefore, inhibition of these adhesion molecules, particularly ICAM-1, has great potential in the treatment of advanced stage cancers [33, 34]. In our study, HE treatment substantially suppressed the TNF-*α*-induced overexpression of ICAM-1, suggesting inhibited adhesiveness of EA.hy926 cells against cytokine (TNF-*α*) response. In agreement with this, migration and invasiveness of endothelial cells with HE treatment was remarkably inhibited compared to TNF-*α* alone exposure. Similarly, Huang and colleagues [35] demonstrated inhibition of TNF-*α*-induced ICAM-1 expression along with inhibition of tumor cell (A549 cells) invasion and MMP-9 expression. Another study showed that targeted disruption of ICAM-1 gene in mice resulted in greater inhibition of choroidal neovascularization with fewer lesions [36]. Since HE inhibits the ICAM-1, we assume that HE may interfere with adhesion of T-cells to ICAM-1, thereby decreasing the invasion and migration of endothelial cells. Owing to the critical role of ICAM-1 expression in cancer pathogenesis, inhibition of ICAM-1 by HE treatment would be a valuable approach to inhibit cancer cell migration and the spread of cancer cells to distant organs, or both.

It has been indicated that TNF-*α*-induced NF-*κ*B activation stimulates the production of MMPs and adhesion molecules in human endothelial cells [8]. Salicylates, widely used anti-inflammatory substances, are shown to inhibit the TNF-*α*-induced ICAM-1 expression by blocking of NF-*κ*B activation [37]. Therefore, we examined the response of NF-*κ*B with HE treatment in the presence or absence of TNF-*α* in EA.hy926 cells. We found that cells incubated with HE significantly attenuated the TNF-*α*-induced nuclear translocation and transcriptional activation of NF-*κ*B followed by the suppression of I-*κ*B degradation. Like other anti-inflammatory drugs, our findings emphasized that HE can act as a potent antiangiogenic substance through downregulation of MMP-9 and ICAM-1 expressions via suppression of NF-*κ*B activation.

Further studies demonstrated that TNF-*α*-induced NF-*κ*B activation and gene expression are mediated by ROS, and these events can be inhibited by the intracellular ROS inhibitor, N-acetylcysteine [9, 38]. NF-*κ*B has two levels of redox regulation: one in the cytoplasm and another in the nucleus. The former involves the phosphorylation of two serine residues on I-*κ*B*α*, which results in its polyubiquitination and subsequent degradation by the 26S proteasome, permitting the unmasking of the nuclear localization signal and the translocation of activated NF-*κ*B into the nucleus. The latter process involves the direct redox modification of specific cysteine residues in the DNA-binding domain of NF-*κ*B in the nucleus [39]. Since TNF-*α* exerts a pleiotropic action on multiple cell functions through the generation of ROS [40], it is possible that HE alters EC function by inhibiting TNF-*α*-induced NF-*κ*B activation via suppression of ROS generation. On the other hand, treatment of polysaccharide protein HEG-5 (*H. erinaceus*) to gastric cancer cells was shown to promote the caspase-mediated apoptosis and cell cycle arrest that are associated with decreased expressions of P13K and AKT [21]. Since activation of P13K/Akt signaling is subjected to oxidative injury and cell survival, it cannot be ruled out that HE-induced inhibition of ROS possibly occurred through modulation of P13K/Akt signaling in EA.hy926 cells. Substantial inhibition of intracellular ROS by HE treatment against TNF-*α*-induction may support this phenomenon.

It has been well documented that several dietary ingredients or phytochemicals possess potent antioxidant properties not only by scavenging the toxic ROS, but also by promoting the transcriptional activation of antioxidant genes [41]. Nrf2, a basic leucine-zipper (bZIP) transcription factor, is involved in induction of various antioxidant genes, including HO-1 and *γ*-GCLC [39]. Previous studies from our laboratories repeatedly confirmed that increased Nrf2 activity is beneficial to protect the cells from oxidative stress [14, 26, 42]. This protection was accompanied by increased expressions of HO-1 and *γ*-GCLC genes and simultaneous inhibition of ROS generation. Furthermore, cells with lack of Nrf2 gene were susceptible to ROS-induced oxidative damage and inflammation [14, 42]. Cells treated with plant-derived chemicals reported to enhance the Nrf2-dependent ARE activity and induce HO-1 and *γ*-GCLC expressions in variety of cells, including endothelial cells [11, 43].

Similarly, endothelial cells incubated with HE showed increased expressions of HO-1 and *γ*-GCLC via Nrf2 activation. HO-1 is a major antioxidant enzyme that plays an essential role in antioxidant defense against toxic free radicals or ROS-induced oxidative destruction in human endothelial cells [44, 45]. These beneficial effects of HO-1 are mediated by the metabolic end products produced from enzymatic degradation of free heme. Biliverdin and its metabolite bilirubin confer potent cellular antioxidant capacities and CO acts as an important signaling molecule with anti-inflammatory and antiapoptotic effects [44, 46]. Besides its host defensive role against oxidative injury, HO-1 also exhibits anti-inflammatory activity in cells and tissues [45]; thus increased HO-1 by HE may explain its crucial role under stimulation. Increased *γ*-GCLC by HE contributed to increase the intracellular GSH levels. GSH is a well-studied thiol-based antioxidant reported to protect the cells from ROS-induced oxidative stress, thereby maintaining the cellular thiol redox status [47]. Nrf2 has been shown to regulate the expression of many thiol-regulating enzymes, including *γ*-GCLC. Increased GSH levels act as a nonenzymatic antioxidant by direct interaction of SH group with ROS or are involved in the enzymatic detoxification reaction for ROS elimination, as a cofactor or coenzyme [48]. These results confirmed that HE can protect the endothelial cells from TNF-*α*-induced ROS generation and inflammation possibly through elevation of cellular antioxidant status.

Several studies claimed that there is a cross talk existing between Nrf2 and NF-*κ*B redox transcriptional factors, and modulation of this cross talk could protect the cells from oxidative damage and inflammation [11]. Our findings support that HE-induced Nrf2 activation and NF-*κ*B suppression were led by decreased angiogenesis in TNF-*α*-stimulated endothelial cells. This antiangiogenic effect of HE possibly occurs through Nrf2 activation (stimulated antioxidant genes) and/or NF-*κ*B inhibition. A recent study showed that increased Nrf2-mediated antioxidant genes and suppressed NF-*κ*B activation by coenzyme Q_0_ resulted in inhibition of the TNF-*α*-induced angiogenesis in EA-hy926 cells [49]. This data implies that both antioxidant and anti-inflammatory actions may control the excessive angiogenesis and protect the cells. As per our knowledge, this is the first study to reveal the underlying Nrf2/NF-*κ*B signaling and ROS inhibition behind the antiangiogenic effects of HE in EA-hy926 cells. Nevertheless, detailed in-depth studies are warranted to confirm the molecular mechanism.

The vital bioactive compounds that existed in* H. erinaceus* may be responsible for most of its pharmacological properties, including antiangiogenic, anti-inflammatory, and antioxidant actions. Crude or purified polysaccharides extracted from the fruiting body of* H. erinaceus* exert antitumor effects primarily by activating various immune system responses [50, 51]. A recent study by Zan and colleagues [21] demonstrated that polysaccharide protein HEG-5 purified from* H. erinaceus* inhibits the gastric tumor cell growth by promoting the cell cycle arrest and apoptosis. Furthermore, novel diterpenoid compounds, erinacines (A–I) isolated from* H. erinaceus*, are regarded to have nerve regenerating property and able to pass through the blood brain barrier to heal on myelin or nerve tissue [19, 52]. In this study, the determined total polyphenols, flavonoids, pentose, and hexose contents in* H. erinaceus* were about 0.08%, 0.01%, 0.8%, and 1.08%, respectively. Although the precise compounds responsible for its antiangiogenic, anti-inflammatory, and antioxidant efficacies have not yet been illustrated, we assume that total polyphenols, polysaccharides proteins, and flavonoids in HE may contribute to most of its pharmacological effects. In nearing future, we have sought to investigate the antiangiogenic effects of specific bioactive compounds isolated from HE.

## 5. Conclusions

Many medicinal mushrooms and herbs have been shown to be the rich source of phytochemicals with chemoprevention potential for various human cancers and inflammatory diseases. The results demonstrate that* H. erinaceus* markedly inhibited the TNF-*α*-induced angiogenesis in human EA.hy926 endothelial cells through downregulation of MMP-9/NF-*κ*B signaling and upregulation of Nrf2-mediated antioxidant genes (Figure 8). These findings added some novel information on the biological activities of* H. erinaceus*, in relevance to suppression of toxic ROS and upregulation of antioxidant genes. However, further* in vivo* studies may provide new therapeutic insights of this potentially beneficial mushroom.
